# The Long and Winding Road (Apologies to the Beatles)

**DOI:** 10.1371/journal.ppat.1005094

**Published:** 2015-08-27

**Authors:** Hank Seifert

**Affiliations:** Department of Microbiology-Immunology, Northwestern University—Feinberg School of Medicine, Chicago, Illinois, United States of America

I was an indifferent high school student at the alternative Miquon School in Philadelphia, Pennsylvania—a bit lazy, suspicious of authority, with the wrong type of friends; but with an analytical and curious mind. Although I didn’t know it at the time, these were good attributes for a future academic scientist. At Beloit College in Wisconsin, I was still a bit lazy but had great, interactive professors who stimulated me to think for myself and to realize that hard work and knowledge had their own rewards. While I found chemistry to be boring, in my junior year I started reading on my own about the cloning revolution that was occurring in the late ‘70s and got really excited about the possibilities. After graduation, I worked at the United States Patent and Trademark Office as a patent examiner for two years, but found the law and bureaucracy not to my liking and, therefore, joined a biophysics/molecular PhD program at Penn State. The only laboratory that I was interested in working in that had openings (for a largely untrained person with a weak undergraduate record) was that of Ron Porter, a new Assistant Professor who was studying homologous recombination processes in *Escherichia coli*. While I failed miserably in my research project for the first 18 months, I found it exciting and challenging, and I learned more in that time than in any other period of my scientific life. It was in graduate school that I began the life-long process of learning how to be a scientist. A seminar by my future postdoctoral advisor, Maggie So, on the initial description of the *Neisseria gonorrhoeae* pilus phase and antigenic variation system, grabbed my attention. It was so cool. After Maggie’s seminar, I asked if she had any openings for postdocs, and when she found out that my research focus was on homologous recombination, she recruited me to the Research Institute of Scripps Clinic in La Jolla, California, for postdoctoral training. There, I learned a lot from Maggie, my collaborator Fred Heffron, many other postdocs, and from the stream of senior researchers who liked to visit La Jolla in the winter. I was hired by Pat Spear at Northwestern University Medical School to start my own research program into *N*. *gonorrhoeae* pathogenesis. As a faculty member, I had to learn more about microbial pathogenesis (for research and teaching); immunology (because it’s important and interesting); how to teach effectively; how to manage a laboratory; how to mentor students, postdocs, and other faculty; and most importantly, how to communicate effectively. I have found this life as an academic researcher to be challenging and extremely fulfilling. I interact daily with a smart, dedicated cadre of younger scientists; have great colleagues; and am part of an international community of dedicated, nice people.

I have been studying the pathogenic *Neisseria*, *N*. *gonorrhoeae* and *N*. *meningitidis*, for over 30 years. These two pathogens—the gonococcus (Gc) or the meningococcus (Mc)—are closely related organisms that colonize distinct physiologic sites within people and cause very different diseases. Gc is the sole causative agent of gonorrhea, which is a sexually transmitted infection. Mc is one of many causative agents of meningitis, but is the main bacterium causing meningitis in teenagers and young adults. These organisms are only found within humans and have evolved from human-specific commensal bacteria. They most often colonize people without causing disease symptoms, but each has gained the ability to cause disease in otherwise healthy individuals. Gc and Mc are important examples of organisms that have crossed the line between a non–disease-causing commensal organism and a true pathogen. The bacterial factors that allow colonization are largely shared between the commensal *Neisseria* and the pathogens, but the pathogens have developed interactions with their host that cause damage. For Gc, it is accepted that the ability to elicit a massive inflammatory response made up primarily of neutrophils in the genital tract has allowed this organism to become a successful pathogen by, presumably, increasing transmission between hosts. This inflammatory response, unfortunately, has the ability to damage cells and tissues in the female reproductive tract. We are interested in understanding how Gc modulates neutrophils to promote pathogenesis. This involvement of neutrophil inflammation in the pathogenesis of Mc meningitis may also be important to disease, but this relationship is not as well established. Another distinguishing characteristic of Gc and Mc is their ability to undergo phase and antigenic variation of important surface structures. They each express at least three diversity generation systems to provide variant subpopulations that can be selected for by functional differences or to escape immune surveillance. Our work on the pilus system investigates how high-frequency variation occurs and how variation can alter or maintain pilus functions. Our research attempts to define specific mechanisms behind the pathogenesis of gonorrhea and meningococcal meningitis, but is not translational in the strict sense of the term. While the mechanisms we uncover could have clinical implications, we not trying to create cures or diagnostics. We are driven by the concept that we need to understand the relationship between us and the microbial world before the knowledge can be applied to improve our health and world.

**Image 1:**
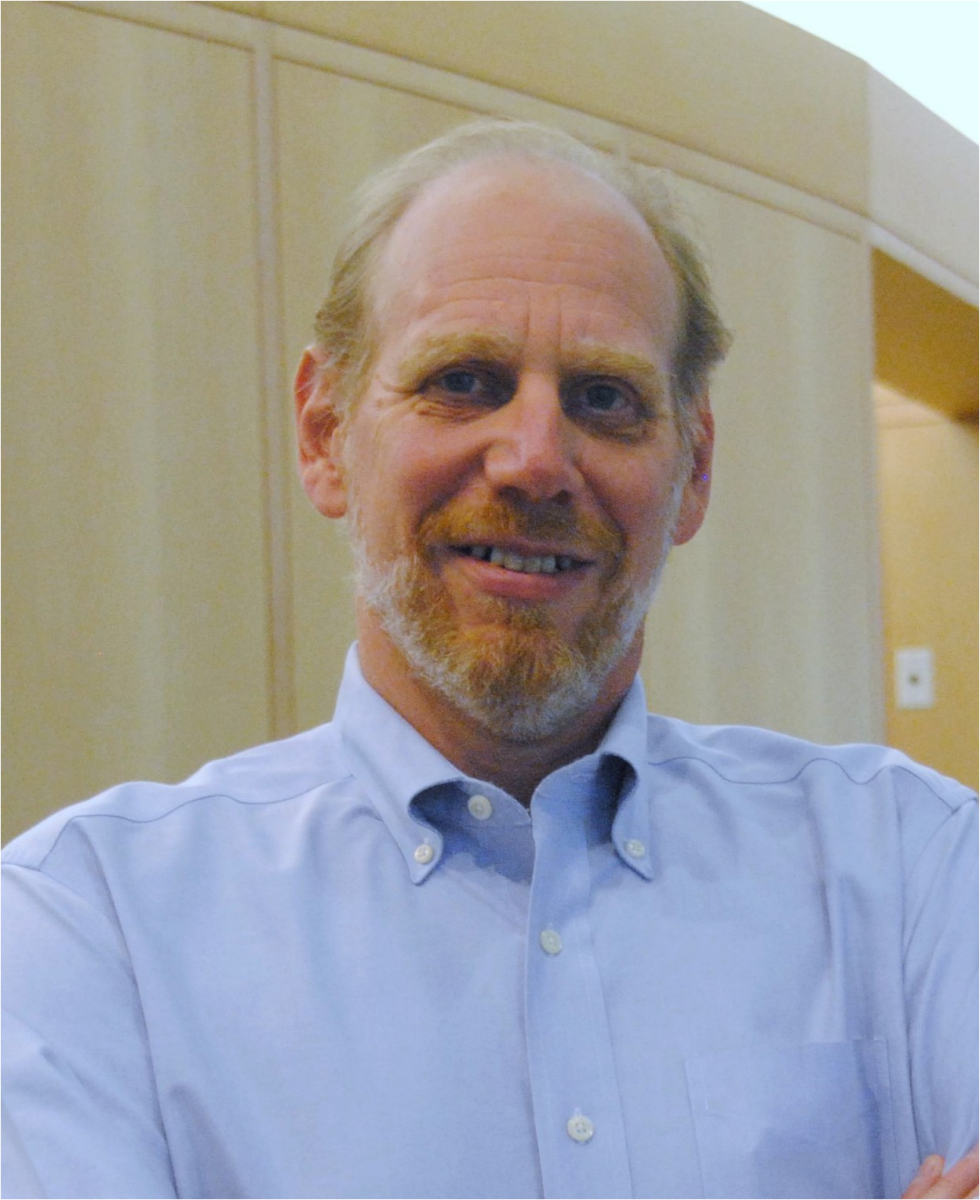
Hank Seifert.

